# Electroacupuncture Reduces Weight in Diet-Induced Obese Rats via Hypothalamic Tsc1 Promoter Demethylation and Inhibition of the Activity of mTORC1 Signaling Pathway

**DOI:** 10.1155/2018/3039783

**Published:** 2018-04-26

**Authors:** Jincheng Leng, Feng Xiong, Junpeng Yao, Xiahuan Dai, Yulei Luo, Maoqing Hu, Lin Zhang, Ying Li

**Affiliations:** ^1^Chengdu University of Traditional Chinese Medicine, Chengdu, China; ^2^Department of Acupuncture, Hospital of Traditional Chinese Medicine, Dazu District, Chongqing, China; ^3^Department of Cardiology, The Third People's Hospital of Chengdu, Chengdu, China; ^4^The Third Affiliated Hospital of Chengdu University of Traditional Chinese Medicine, Diabetes Mellitus Prevention and Control Center of Sichuan Province, Chengdu, China

## Abstract

**Subject:**

The study aimed to investigate the mechanism of electroacupuncture reducing weight via tuberous sclerosis complex 1 (Tsc1) promoter methylation, inhibiting the mammalian target of rapamycin complex 1 (mTORC1) pathway.

**Materials and Methods:**

Male Sprague-Dawley rats were divided into chow-fed group (chow group) or high-fat diet group (HF group) for 14 weeks. The obesity rats in HF group were randomly divided into electroacupuncture group (EA group) and diet-induced obesity (DIO) group, which received EA stimulation on bilateral ST25, RN12, SP6, and ST36 for 4 weeks or no further treatment, respectively. Methylation of the Tsc1 gene promoter and expression of agouti-related protein (AgRP), neuropeptide Y (NPY), and proopiomelanocortin (PoMC) were detected at the 18th week.

**Results:**

At week 18, weight, body fat, and the body fat rate in DIO group were significantly higher than those of the chow and EA group. Compared with the chow group, the DIO group had increased methylation of the Tsc1 gene promoter and expression of mTORC1, AgRP, and NPY gene and decreased PoMC in the hypothalamus; after EA, methylation of the Tsc1 gene promoter, mRNA, and protein of the mTORC1 and expression of AgRP and NPY gene decreased and PoMC increased significantly.

**Conclusions:**

Our study could shed light on the potential pathway where EA exerts effects on the mechanism of EA treatment for obesity through the hypothalamic Tsc1 promoter demethylation and inhibition of the activity of mTORC1 signaling pathway.

## 1. Introduction

Obesity can cause many complications, such as cardiocerebral disease, type 2 diabetes mellitus, hypertension, hyperlipidemia, and tumor, which has severely threatened human health and life and attracted much attention in many countries [1, 2]. Obesity has globally reached epidemic proportions, as a result of being overweight or obese, at least 2.8 million people die each year. In 2014, data showed that over 1.9 billion people aged 18 years or older were overweight, including 600 million people with obesity, and the number of overweight or obese people showed a continuous increase trend [3]. In men, age-standardized prevalence of obesity grew from 3.2% in 1975 to 10.8% in 2014 and from 6.4% to 14.9% in women. Prevalence of severely obese was 2.3% and 5.0% in men and women, respectively, and the percentage of morbid obesity was 0.64% and 1.6% (1.3–1.9). Data showed that the incidence of obesity in male was 43.2 million people and the number of women obese was 46.4 million in China, which was the first in the world [4]. Therefore, obesity has been an urgent issue for us to resolve.

To date, many studies have shown that epigenetics is closely associated with obesity [5, 6] and is likely to be a major cause for obesity. Therefore, exploring the mechanism for simple obesity from an epigenetic perspective may provide a breakthrough and will help find new targets of obesity treatment. DNA methylation is one major mode of epigenetics of obesity [7, 8]. Previous experimental studies by our research group found that the promoter region of the tuberous sclerosis complex 1 (Tsc1) gene in rat hypothalamus had an obesity-related methylation site; the obesity rat Tsc1 promoter appeared to be at a high methylation state and its downstream mammalian target of rapamycin (mTOR) gene expression was upregulated, which may be attributed to a weakened inhibitory effect on the mTOR gene as a result of an increased methylation of the Tsc1 promoter, thereby increasing appetite and food intake and finally causing obesity [9]. These findings indicated that Tsc1 controlled the downstream mTOR signaling pathways partly by receiving many upstream stimulations (i.e., intracellular or extracellular growth factors, environmental change signals, energies, and nutrients) and played a pivotal role in the process of growth, differentiation and migration of cells, development of embryos, and metabolism of the body, while a malfunction in the process will cause tumor, obesity, or other metabolic diseases [10–13].

To date, there has not yet been an effective therapy for obesity. Therefore, searching for effective treatments and mechanisms is a hot yet difficult issue. Obesity is one of common and preponderant diseases treated with acupuncture. Many ancient and modern literatures and clinical trials have verified that acupuncture has a good therapeutic efficacy on simple obesity [14–16]. In this study, we fed SD rats with high-fat feed and then we treated them with electroacupuncture and measured the expression levels of obesity-related factors in the rat hypothalamus after the experiment, further elucidating the molecular mechanism of acupuncture for weight loss.

## 2. Materials and Methods

### 2.1. Animals and Animal Care

All experimental procedures were approved by Institutional Animal Care and Use Committee of Chengdu University of Traditional Chinese Medicine for animal research. SPF-grade SD male rats that had weights of 70~90 g and had just been weaned (three to four weeks of age) were chosen. The rats were all provided by Chengdu Dashuo Biotechnology Company, Ltd., with an animal production license number of SCXK (SiChuan) 2013-2024. After arrival, the experimental animals were fed at a density of five rats per cage, during the first week with standard laboratory water and chow ad libitum, and then allowed to take food and drink water freely under a 12 h natural light-dark cycle at a temperature in the range of 22°C–24°C, and ventilation was conducted on a regular time basis. The relative humidity was 50%–70%. Rat cages were cleaned and drinking flasks were rinsed every day, and the feeds were ensured to be clean and fresh. Animals were divided into high-fat (HF) and normal feed group (chow group) group randomly. The HF group of rats were fed with the high-fat feed (fat 20%, glucose 10%, dry powder of yolk 10%, standard chow 60%, 492.8 kcal/100 g) [17] in enough amount for 14 weeks, while the chow group of rats (*n* = 18) were fed with normal feeds in enough amount for 14 weeks. Food and water were provided ad libitum. The rat weights were measured once a week. After all the rats were fed using the above methods for 14 weeks, they were fasted but allowed to drink water for 12 h. According to the literature, when fasted, rats of the model group were considered to be modelled successfully if they were in a good health condition and had a weight 20% higher than the average weight of the chow group, and such rats were used as diet-induced obesity (DIO) rats [18]. Then these DIO rats were randomly were divided into electroacupuncture therapy (EA) group (*n* = 18) and DIO group (*n* = 18), which received EA stimulation for 4 weeks and no further treatment, respectively.

### 2.2. Electroacupuncture Therapy

According to the literatures, four acupuncture points, Tianshu (ST25) (5 mm lateral to the navel), Zhongwan (CV12/RN12) (20 mm above the umbilicus on the midline of the upper abdomen, with a needle inserted at 2 mm) [19], Sanyinjiao (SP6) (located in the posterior border of the tibia, 0.5 cm above the medial malleolus) [20], and Zusanli (ST36) (between the tibia and the fibula approximately 5 mm lateral to and below the anterior tubercle of the tibia) [21], were selected. Disposable acupuncture needles with a size of 0.18 mm × 13 mm were used. After routine sterilization, the needles were inserted into the acupuncture points and slightly twisted to Deqi, with each needle handle connected to a SDZ-II Huatuo Electroacupuncture Instrument (Suzhou Medical Appliance Factory, China). The stimulation parameters included a disperse-dense wave of 2/15 Hz, a treatment duration of 30 min/d × 30 d, with a 2-d break after every 5-d treatment. The nonacupuncture group of rats were anchored with the same holder but were not subjected to any intervention therapy.

### 2.3. Body Weights, Body Fats, and Percentage of Body Fat of the Rats

The body weight of each rat in each group was measured with an electronic scale and recorded on a regular time basis every week before intervention therapy; after intervention therapy, the body weight and body fat of each rat in each group were measured with an electronic scale, and then the body fat rate was calculated:

(1) The  body  fat g=greater  omentum  fat+perirenal  fat+epididymal  fat,The  percentage  of  body  fat %=the  body  fatthe  body  weight×100%.

### 2.4. Expression of Methylation of the Tsc1 Gene Promoter and mTORC1, Agouti-Related Protein (AgRP), Neuropeptide Y (NPY), and Proopiomelanocortin (PoMC)

The weight of each rat in each group was measured on the day when the experiment was completed. Next, all the rats were fasted for 12 h and then were intraperitoneally anaesthetized with of 7% chloral hydrate (0.5 ml/100 g) on the second day. After anesthesia, they were killed by cervical dislocation, and then their heads were cut off. Their brain tissues were immediately isolated on an ice bath. The hypothalamus was cleaned with normal saline and then placed in a liquid nitrogen container for use. After all the brain specimens were preliminarily collected, they were subjected to a further extraction. With western blot and RT-PCR, the methylation of the Tsc1 gene promoter and the expression of mTORC1, NPY, AgRP, and PoMC in the arcuate nucleus were measured.

#### 2.4.1. Measurement of Tsc1 Promoter Methylation

Methylated DNA immunoprecipitation (MeDIP) PCR was used to detect the DNA methylation. DNA was extracted from hypothalamus tissues using the Micro DNA kit (TianGen Biotech (Beijing) Co., Ltd.) according to the manufacturer's instructions. Gene DNA was interrupted by ultrasound. CGP DNA was extracted using EpiMark Methylated DNA Enrichment Kit (New England Biolabs). Tsc1 promoter, total of 511 bp, from the 15628th base to the 16139th base of the Tsc1 gene sequence, was searched in NCBI database and found 11 CpG island sites. Primer of Tsc1 promoter is listed in Table 1. The qPCR program was set to 95°C for 30 s and 40 cycles of 95°C for 5 s, 55°C for 30 s, 72°C for 30 s. Value of CT was analyzed with the Sequence Detection software version 1.2.3 (Applied Biopsy Stems Company). Levels of mRNA expression were presented as the form of 2^−ΔΔCt^ from at least three independent experiments after being normalized to *β*-actin.

#### 2.4.2. Western Blot

Hypothalamus was mixed with RIPA buffer (RIPA : Cocktail = 100 : 1),and centrifuged at 4°C, 12000 rpm for 10 min; supernatants were boiled for 5 minutes with 5x SDS-PAGE Loading (volume 4 : 1). SDS-PAGE was prepared with deionized water, 1.5 M Tris-HCl (PH 8.8), 10% SDS, 10% APS and TEMED. Protein samples (100 *μ*g) were transferred to a nitrocellulose membrane (Hybond, USA) with 200 V voltage for 2 hr and blocked with 5% 5% BSA for 2 hr and then washed with TBST 3 times, 5 min/time. After blocking, the membrane was incubated with the primary antibody (mTORC1 : 1 : 1000, abcam, UK) overnight at 4°C. After incubation, the membranes were washed 3 times, 5 min/time, followed by incubation with the secondary antibody (1 : 5000) for 2 hr and washed 3 times, 10 min/time. Protein bands were analyzed using Gel image analysis and imaging system (JunYi, Beijing, China).

#### 2.4.3. RT-PCR

Total RNA was extracted from hypothalamus tissues using the Trizol (Invitrogen, USA) according to the manufacturer's instructions. Complementary DNA from the RNA was synthesized using the complementary DNA reverse transcription kit (TaKaRa Bio, Dalian, China). RT-PCR primers are listed in Table 2. mRNA expression of mTORC1, AgRP, NPY, PoMC, and *β*-actin (internal control) was determined according to the TaqMan gene expression kits and PCR reagents. The reaction volume was 20 ul with 0.8 ul PCR Forward Primer (0.4 *μ*M), 0.8 ul PCR Reverse Primer (0.4 *μ*M), and 2.0 ul cDNA. The qPCR program was set to 95°C for 30 s and 40 cycles of 95°C for 5 s, 55°C for 30 s, and 72°C for 30 s. Value of CT (Threshold cycle) was analyzed with the Sequence Detection software version 1.2.3 (Applied Biopsy Stems Company). Levels of mRNA expression were presented as the form of 2^−ΔΔCt^ from at least three independent experiments after being normalized to *β*-actin.

### 2.5. Statistical Analysis

SPSS20.0 was used for statistical analysis. All the results were expressed as mean ± SD. The data were assessed by one-way ANOVA if the assumptions for parametric analysis fulfilled the condition. Otherwise, a nonparametric Kruskal-Wallis Test was used. All bar graphs were performed using the GraphPad Prism version 5.00 software (San Diego, California, USA). *P* < 0.05 was considered as statistically significant.

## 3. Results

### 3.1. Effects of Electroacupuncture on the Weight, Body Fat, and the Percentage of Body Fat

**Figure 1:**
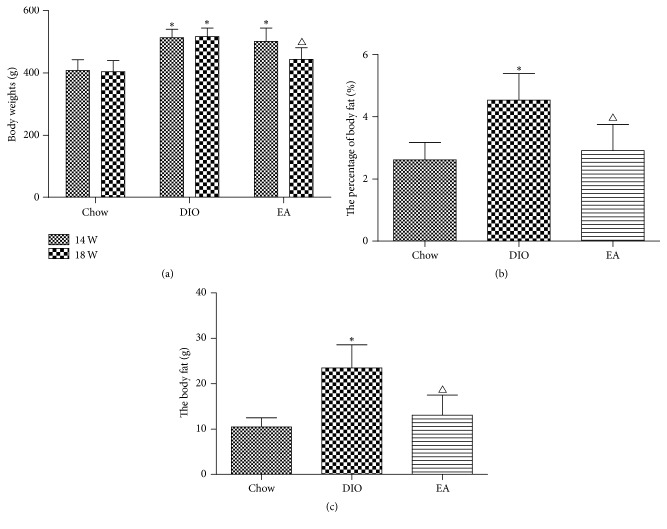
Change of body weight gain and body fat before and after electroacupuncture. (a) Body weights change at 14 and 18 weeks in different groups (*n* = 18). At week 14, body weight was higher in DIO and EA than that of the chow group. At week 18, body weight in EA group was significantly lower than that of the DIO group. (b) The percentage of body fat in each group at week 18. (c) Body fat in each group at week 18. ^*∗*^*P* = 0.001 DIO versus chow; ^△^*P* = 0.001 EA versus DIO.

**Figure 2:**
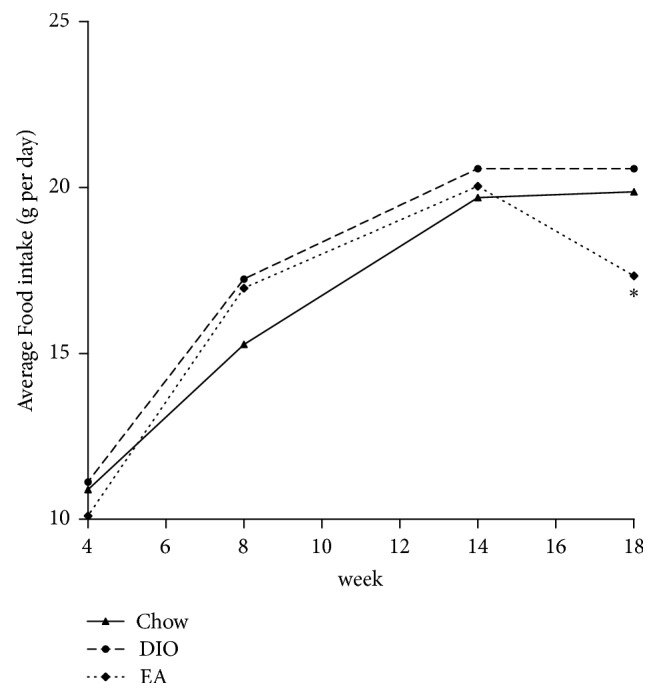
Average food intake (g per day) in each group. At week 18, ^*∗*^*P* = 0.001 EA versus DIO.

At week 14, the weight of the chow group (fed by ordinary feed) was 408.00 ± 34.75 g, while the average weights of the DIO group and the EA group (both fed by high-fat feed) were 513.44 ± 26.61 g and 501.44 ± 42.99 g, respectively, both significantly higher than that of the chow group. There was no difference in weight between the DIO group and the EA group before the treatment. At week 18 after the experiment, the average weight of the chow group was 404.11 ± 35.58 g, while the average weight of the DIO group was 516.71 ± 27.61 g, significantly higher than that of the chow group, and the average weight of the EA group (443.71 ± 37.52 g) was also significantly higher than that of the chow group but significantly lower than that of the DIO group (Figure 1, Table 3), the extent of body weight decrease by EA was 11.47%. At the same time, the body fat and the percentage of body fat in each group were decreased in the following order: the DIO group > the EA group > the chow group (Figure 1, Table 3). The results showed that EA could improve both the body fats and the percentage of body fat of rats, thereby alleviating obesity.  Figure 2 showed the food intake in each group at different week; at 18 week, the average food intake per day was significantly lower in DIO group than that in EA group (*P* = 0.001), which suggested that EA can decrease appetite.

### 3.2. Results of DNA Methylation of the Tsc1 Promoter Gene in the Rat Hypothalamus of Each Group

**Figure 3:**
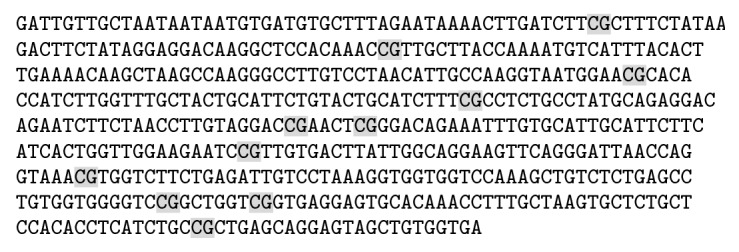
Methylation sites of Tsc1 promoter gene.

**Figure 4:**
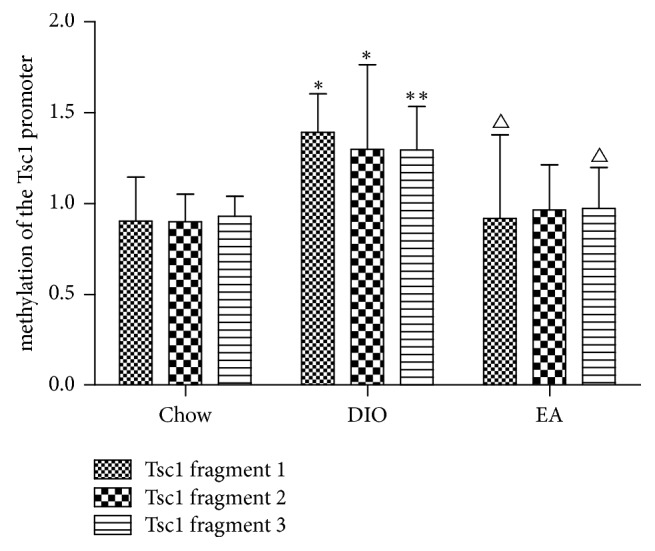
Methylation of Tsc1 promoter gene fragments 1, 2, 3 (*n* = 6 in each group). ^*∗*^*P* < 0.05, DIO versus chow in fragments 1, 2. ^*∗∗*^*P* < 0.01 DIO versus chow in fragment 3; ^△^*P* < 0.05 EA versus DIO in fragments 1, 3.

Mori's study showed that Tsc1 gene activated the mTORC1 activity, further influencing the factors that regulated appetite and thus increasing food intake and weight [22]. According to the NCBI (http://www.ncbi.nlm.nih.gov/gene/60445), the sequence of methylation sites of Tsc1 promoter gene with the size of 511 bases was from the beginning of 15628th base to the 16139th base. The labelled CG was the methylation sites (Figure 3). As seen in Figure 4, as compared with the chow group, the DIO group had increased methylation of the Tsc1 gene segments 1, 2, and 3; the methylation level of the Tsc1 gene segments 1 and 3 after electroacupuncture was decreased, significantly lower than that in the DIO group, and not statistically different from that in the chow group; the methylation level of the Tsc1 gene segment 2 after electroacupuncture was decreased but was not statistically different from those in both the DIO group and the chow group.

### 3.3. mRNA and Protein Expression of the mTORC1 Gene in the Rat Hypothalamus of Each Group

**Figure 5:**
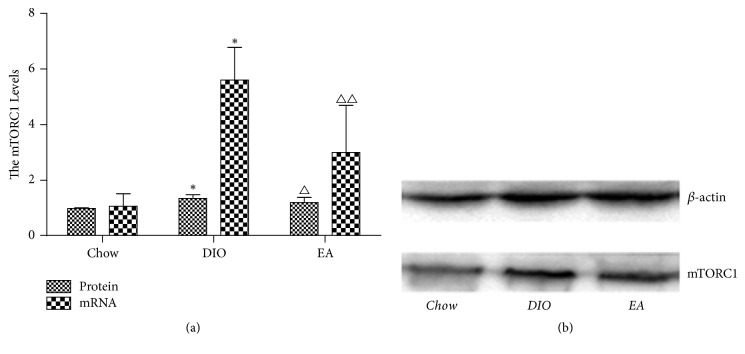
Effects of EA on mTORC1 in hypothalamus (*n* = 6 in each group). (a) mRNA and protein expression of the mTORC1 gene in hypothalamus. ^*∗*^*P* < 0.01 DIO versus chow; ^△^*P* < 0.05  ^△△^*P* < 0.01 EA versus DIO. (b) Western blots in each group to show mTORC1 protein content.

mTORC1 in the hypothalamus primarily functions in the central hypothalamic neurons, thus influencing food intake and causing a change in weight. As seen in Figure 5, mRNA and protein of the mTORC1 gene in the rat hypothalamus of the chow group had a certain amount of expression; as compared with the chow group, the DIO group had more significantly increased mRNA and protein of the mTORC1 gene in the hypothalamus; after electroacupuncture, mRNA and protein of the mTORC1 gene in the hypothalamus of the EA group were obviously decreased, significantly lower than those in the hypothalamus of the obesity group but higher than those in the hypothalamus of the chow group.

### 3.4. Expression of PoMC, NPY, and AgRP mRNA in the Rat Hypothalamus of Each Group

**Figure 6:**
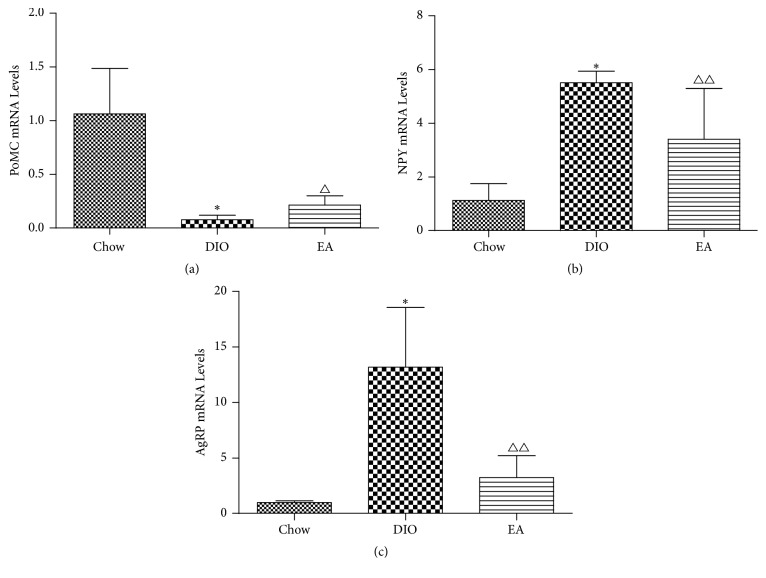
Effects of EA on PoMC, NPY, and AgRP mRNA in hypothalamus (*n* = 6 in each group). Expression of PoMC (a), NPY (b), and AgRP (c) mRNA in hypothalamus. ^*∗*^*P* < 0.01 DIO versus chow; ^△^*P* < 0.05, ^△△^*P* < 0.01 EA versus DIO.

Rat appetite-related factors influenced food intake by rats and then caused a change in the body weight. As seen in Figure 6, as compared with the chow group, the DIO group had more significantly increased mRNA expression levels of the AgRP gene and the NPY gene in the hypothalamus; after electroacupuncture, the mRNA expression levels of these two genes were significantly decreased and lower than those in the DIO group, and the mRNA expression level of the AgRP gene in the hypothalamus of the EA group was higher—but without statistical difference—than that in the hypothalamus of the chow group, and the mRNA expression level of the NPY gene in the hypothalamus was significantly higher than that in the chow group.

Compared with the chow group, the DIO group had a more significantly decreased mRNA expression level of the PoMC gene in the hypothalamus; after electroacupuncture, the mRNA expression level of the PoMC gene in the hypothalamus of the EA group was significantly decreased, higher than that in the hypothalamus of the DIO group, and lower than that in the hypothalamus of the chow group. The results indicated that electroacupuncture decreased the expression levels of the two food intake factors, NPY and AgRP, suppressed food intake, and increased PoMC expression, causing weight loss of the experimental animals.

## 4. Discussion

Our study investigates the mechanism of electroacupuncture reducing weight. Obesity rats have increased methylation of the Tsc1 gene promoter and expression of mTORC1, AgRP, and NPY gene and decreased PoMC in the hypothalamus; EA treatment can lower body weight with the extent of body weight decrease being 11.47% and body fat and the body fat rate in DIO rats by demethylation of the Tsc1 gene promoter, decreasing expression of mTORC1, AgRP, and NPY and increasing PoMC.

mTOR signaling pathways is involved in energy metabolism. When the rats were fed, the activity of mTOR in the arcuate nucleus of the hypothalamus increased, and the expression of the downstream target enzyme also increased, which indicates that the energy status of the body can affect the activation of mTOR. Subsequently, when the rats were starved for 48 h, the activity of mTOR decreased significantly, while the rats were fed, the hypothalamus mTOR activity increased again, thereby clarifying the important role of hypothalamic mTOR in the energy perception of the body [23]. Cota et al. found the similar results [24]. TSC1, one of the protein products of the tumor suppressor TSC gene, has GTPase activity and is an important suppressor of upstream of the TOR signaling pathway [22, 25]. Tsc1 deletion mice developed hyperphagia, increased fat pads, and obesity and displayed increased activity of mTOR signaling [22]. These findings indicate that the absence of Tsc1, an inhibitor of mTOR signaling in the thalamus, leads to a loss of control of the feedback system of the mTOR signaling pathway, suggesting that hyperactivation of mTOR signal can make animals eat more nutrients, causing organisms to suffer from obesity [22].

Epigenetics refers to heritable changes that do not involve changes in DNA sequence, the most common form is DNA methylation [26]. DNA methylation is catalyzed by DNA methyltransferase transferring the methyl group of S-adenosylmethionine (SAM) to the fifth carbon atom of cytosine to generate 5-methylcytosine chemical modification process. Most of studies focused on hypermethylation of CpG Islands. Our previous experimental studies found that the promoter region of the Tsc1 gene in rat hypothalamus appeared to be at a high methylation state and its downstream mTOR gene expression was upregulated, thereby increasing appetite and food intake and finally causing obesity [9]. In this study, we found the similar results and the subtype of mTOR, mTORC1 unregulated. Furthermore, we found that EA can reduce weight of obesity rat and, at the same time, the hypothalamic Tsc1 promoter demethylation and inhibition of the activity of mTORC1 occurred. The performance of the signal pathway might play a role in the effect of EA in reducing weight gain of obesity rat. However, further study with the inhibition of hypothalamic Tsc1 promoter demethylation is needed to investigate the phenomenon. Transcriptional epigenetic alterations in the adipose tissue were found in women with polycystic ovary syndrome (PCOS); EA treatment leads to an acute and global effect on the methylome and the transcriptome directly [27]. Wen and Lee showed that EA treatment decreased fat mass and adipocyte size accompanied by decreased adipose tissue inflammation through hypoxia-inducible factors-1*α*-dependent pathways [28]. These studies may provide evidence for the influence of EA on alterations of epigenetic and gene change. Qi is considered as the basis of acupuncture and is called an energy force traveling throughout our body along “meridians” or special pathways [29]. The meridians come to the specific surface of the skin where they are considered as acupuncture points. Qi is the media between them. Epigenetic mechanisms, such as DNA methylation and histone modification, provide a plausible explanation for nongenetic disease transmission [30, 31]. Maybe the Qi is the association between acupuncture and DNA methylation.

Obesity is a disease of abnormal weight gain caused by excessive fat accumulation in the body when calorie intake is more than calorie consumption, without any known etiology. It is currently believed that obesity is caused by a coaction of many factors, such as genetics and environment. Obesity often causes many diseases, for example, diabetes mellitus, metabolic syndrome, and cardiocerebral disease. Many studies even showed that the incidence of cancer in obesity patients was higher than that in normal population [32–35]. From a microcosmic aspect, the weight of mammals and human is subjected to dual regulation by the neural system and the endocrine system, with the center of the both systems being located at the hypothalamus. The hypothalamus mains energy metabolism at a dynamic balance in the body by regulating appetite, allowing the weight to fluctuate in a small range [36–38]. Many hypothalamic nucleuses, for example, ventromedial nucleus (VMH) [39], arcuate nucleus (ARC) [40, 41], and paraventricular nucleus (PVN) [42], participate in appetite regulation, are involved in producing and receiving factors that regulate appetite, influence energy consumption rate, and regulate the secretion of hormones related to energy storage, playing a pivotal role in energy balance and body weigh regulation. Some studies have indicated that NPY and AgRP promote appetite, whereas PoMC and cocaine-and amphetamine-regulated transcript (CART) inhibit appetite [43–46].

To date, the major methods controlling obesity include diet, exercise, pharmacotherapy, and surgery [47, 48]. Currently, the mechanism of obesity treatment with acupuncture is studied primarily with animal experiments, but the exact mechanism remains unknown. It is widely accepted that acupuncture-induced weight loss is achieved through the regulation of the neural system, primarily through the stimulation of feeding center in the hypothalamus; with respect to the regulation of the endocrine system, acupuncture can improve the high insulin resistance and leptin resistance of obesity patients and can change carbohydrate metabolism, fat metabolism, and hormone levels, thereby facilitating lipolysis and excretion and finally resulting in weight loss. Treatment of simple obesity with acupuncture has a very complex mechanism, which involves many systems, such as the neural and endocrine system. The effect of acupuncture on the neural system is as follows: the center regulating food intake and feeding behaviors of mammals and humans is located in the hypothalamus [49], and the mechanism of acupuncture treating obesity is associated with hypothalamic regulation. The hypothalamic neurons can secrete two appetite-related hormones: one is NPY facilitating appetite and the other is *α*-melanophore-stimulating hormone (*α*-MSH), and the precursor of the latter one is PoMC. The hypothalamic ARC dominates the secondary neurons of these two polypeptide receptors. As suggested by some studies, the reason for electroacupuncture inhibiting appetite and reducing body weight was likely to be that electroacupuncture controlled the expression of hypothalamic NPY and *α*-MSH/PoMC, resulting in a decreased expression level of the former and an elevated expression level of the latter, which was consistent with the result of previous relevant animal experiments. Moreover, some studies observed that the CART of the hypothalamic ARC was able to inhibit appetite, and electroacupuncture was able to elevate the CART expression level in the ARC [50], indicating that by regulating the appetite-related polypeptides in the hypothalamic ARC, electroacupuncture decreased appetite, and reduced food intake so as to regulate energy balance. The effect on endocrine is as follows: the two hormones of leptin and insulin had a strong inhibitory effect on food intake; as signals of fat and energy metabolism, these two hormones firstly acted on the hypothalamic ARC by crossing the blood-brain barrier because their receptors were mainly concentrated in the ARC neurons. Relevant experiments verified that neurons with the receptors of leptin and insulin simultaneously had expression of the gene PoMC, CART, NPY, and AgRP. This experimental study revealed that the levels of hypothalamic leptin and insulin in the cerebral blood of the obesity rats were significantly lower than those in the cerebral blood of the normal rats, whereas the result of those levels in the peripheral blood was opposite [51]. The stimulation of electroacupuncture significantly reduced the plasma leptin level and simultaneously elevated the level of hypothalamic serum leptin in the obesity rats [52]. As indicated by the above results, acupuncture may increase the binding of leptin and insulin with their receptors in the hypothalamus ARC so as to generate a series of biological effects, namely, an enhancement of the anorexic activity of PoMC and CART or an inhibition of the appetite-gain activity of NPY and AgRP, causing a decrease in food intake and an increase in energy consumption and thereby eventually achieving a weight loss effect.

## 5. Conclusions

With an increase of the methylated modification of the Tsc1 gene promoter in the rat hypothalamus, the activity of downstream mTORC1 was enhanced, which further affected food intake and ultimately resulted in obesity. The treatment of obesity with EA, which was employed to reduce methylation Tsc1 and inhibit the activity of mTORC1, thereby controlling appetite-regulating factors (e.g., NPY, AgRP, and PoMC) and mitigating obesity. This study could shed light on the potential pathway that EA exerts effects on the mechanism of EA treatment for obesity.

## Figures and Tables

**Table 1:** Primer of Tsc1 promoter.

Gene	Forward primer	Reverse primer	Product size (bp)
Tsc1 promoter fragment 1	GGCTTGAACTGTGGTTACTGATGGA	GCAACGGTTTGTGGAGCCTTGTC	210
Tsc1 promoter fragment 2	AGGCTCCACAAACCGTTGCTTACCA	CCACCACAGGCTCAGAGACAGCTT	342
Tsc1 promoter fragment 3	ACTGGTTGGAAGAATCCGTTGTGAC	GGTGTGGAGCAGAGCACTTAGCA	178

**Table 2:** Primer used in RT-PCR.

Gene	Forward primer	Reverse primer	Product size (bp)
mTORC1	agaggaccagcagcacaagcagga	tggtggcagtggtggtggcatt	299
AgRP	agacagcagcagaccgagcagaaga	gccagtacctagcttgcggcagtag	218
NPY	cgtgtgtttgggcattctggctgag	tggtgggacaggcagactggtttca	325
PoMC	tgatggcttggagcacgtcctggag	gcgtctggctcttctcggaggtcat	137
*β*-Actin	gaagatcaagatcattgctcct	tactcctgcttgctgatcca	111

**Table 3:** Weight, body fat, and the percentage of body fat in each group.

Group	*n*	Weight (g)		Body fat (g)	Percentage of body fat (%)
		Week 14	Week 18		
Chow	18	408.00 ± 34.75	404.11 ± 35.58	10.49 ± 2.034	2.62 ± 0.55
DIO	18	513.44 ± 26.61^*∗*^	516.71 ± 27.61^†^	23.50 ± 5.081^†^	4.53 ± 0.85^†^
EA	18	501.44 ± 42.99^*∗*^	443.71 ± 37.52^△^	13.09 ± 4.447^△^	2.91 ± 0.84^△^

Data were presented as mean ± SD. At week 14, ^*∗*^*P* = 0.001 DIO, EA versus chow. At week 18, ^†^*P* = 0.001 DIO versus chow; ^△^*P* = 0.001 EA versus DIO.
